# *Convolvulus pluricaulis* Choisy’s Extraction, Chemical Characterization and Evaluation of the Potential Effects on Glycaemic Balance in a 3T3-L1 Adipocyte Cell Model

**DOI:** 10.3390/nu15071727

**Published:** 2023-03-31

**Authors:** Elisabetta Melloni, Silvia Grassilli, Arianna Romani, Erika Rimondi, Annalisa Marcuzzi, Enrico Zauli, Paola Secchiero, Guglielmo Paganetto, Alessandra Guerrini, Gianni Sacchetti, Massimo Tacchini

**Affiliations:** 1 Department of Translational Medicine and LTTA Centre, University of Ferrara, 44121 Ferrara, Italy; elisabetta.melloni@unife.it (E.M.); erika.rimondi@unife.it (E.R.); paola.secchiero@unife.it (P.S.); 2 Department of Translational Medicine, University of Ferrara, 44121 Ferrara, Italy; silvia.grassilli@unife.it (S.G.); annalisa.marcuzzi@unife.it (A.M.); enrico.zauli@edu.unife.it (E.Z.); 3 Department of Environmental and Prevention Sciences and LTTA Centre, University of Ferrara, 44121 Ferrara, Italy; 4 Department of Life Sciences and Biotechnology (SVeB), UR7 Terra&Acqua Tech, University of Ferrara, 44121 Ferrara, Italy; guglielmo.paganetto@unife.it (G.P.); alessandra.guerrini@unife.it (A.G.); gianni.sacchetti@unife.it (G.S.); massimo.tacchini@unife.it (M.T.)

**Keywords:** *Convolvulus pluricaulis* Choisy, 3T3-L1, adipocyte differentiation, PPARγ, GLUT-4

## Abstract

*Convolvulus pluricaulis* (CP) is a common Indian herb, largely employed in Ayurvedic medicine and known for its neuroprotective and neuroinflammatory action. Its effectiveness against several pathologic/sub-pathologic conditions is widely accepted, but it is not yet completely chemically characterized. In recent years, several researchers have pointed out the involvement of CP and other Convolvulaceae in lipidic and glucidic metabolism, particularly in the control of hyperlipidaemia and diabetic conditions. In this scenario, the aim of the study was to chemically characterize the medium polarity part of the CP whole plant and its fractions and to shed light on their biological activity in adipocyte differentiation using the 3T3-L1 cell model. Our results demonstrated that the CP extract and fractions could upregulate the adipocyte differentiation through the modulation of the nuclear receptor PPARγ (Peroxisome Proliferator-Activated Receptor γ), broadly recognized as a key regulator of adipocyte differentiation, and the glucose transporter GLUT-4, which is fundamental for cellular glucose uptake and for metabolism control. CP also showed the ability to exert an anti-inflammatory effect, downregulating cytokines such as Rantes, MCP-1, KC, eotaxin, and GM-CSF, which are deeply involved in insulin resistance and glucose intolerance. Taken together, these data suggest that CP could exert a potential beneficial effect on glycemia and could be employed as an anti-diabetic adjuvant or, in any case, a means to better control glucose homeostasis.

## 1. Introduction

*Convolvulus pluricaulis* Choisy (CP) is a widespread plant in northern India which commonly grows on the roadside; it is part of a plethora of important herbs traditionally used in Ayurvedic medicine as therapeutic agents [1]. The Ayurvedic pharmacopoeia of India considers the use of the whole plant of CP [1]. In particular, CP is already broadly known for its neuroprotective properties and is associated, for example, with depression syndromes [2] or cerebral reperfusion injury [3]; however, it is also known for its ameliorative effect on neurodegenerative disorders such as Alzheimer’s disease due to its ability to reduce neuroinflammation [4,5]. In in vitro or in vivo models, CP whole extracts have also been shown to be effective against a variety of pathologic or sub-pathologic conditions, such as ulcers, viral and bacterial infections, and liver toxicity [1].

Recently, the interest in CP has been directed towards its role in the regulation of lipid abnormalities in hyperlipidaemic and diabetic in vivo models [6,7]. Indeed, a methanolic extract of CP has been demonstrated to be effective in the control of several lipid parameters in a hyperlipidaemic animal model and to possess significant hypolipidemic activity in a streptozotocin-induced diabetic rat model. Rezq et al. [8] demonstrated that *Convolvulus hystrix* Vahl, which is within the family of Convolvulaceae, exerted a significant lowering effect on the serum glucose concentration in a rat model, in addition to its hypolipidemic activity on triglycerides and high-density lipoproteins. The importance of this new perspective is strictly correlated with the interplay between diabetes, hyperlipidaemia, and cardiovascular disorders [9], and the importance of controlling glycaemia and fat parameters to reduce cardiovascular morbidity and mortality is by now accepted worldwide. In this respect, preclinical studies have already shown the effectiveness of herbal preparations containing CP in the treatment of hypertension [10]. This effect is attributable to the ability to reduce the levels of non-esterified fatty acids that represent a fundamental component of the pathogenesis of cardiovascular diseases [11].

A high concentration of plasma lipids physiologically induces the activation of the intracellular receptors that regulate the metabolism, storage, and even the transport of lipids. The study of these receptors at the pharmacological level is very important as they represent specific targets in lipid metabolism and adipogenesis. In this context, several studies have highlighted the role played by the intracellular receptor PPARγ (Peroxisome Proliferator-Activated Receptor γ), a member of the nuclear receptor superfamily which is known to be a key regulator of adipocyte differentiation and, consequently, of lipidic and glycaemic balance [12]. 

Its importance in adipose tissue as a fundamental regulator of the stromal macrophage function, as well as of the adipocytes, is by now extremely clear since it has been demonstrated that PPARγ inhibits the activation of macrophages and their cytokine production and that it exerts a secondary positive effect on insulin signalling and, finally, on metabolic control [13]. Moreover, PPARγ is increasingly referred to as an anti-inflammatory gene, playing an important role in regulating inflammation in different diseases (e.g., ulcerative colitis or multiple sclerosis) [10]; in some acute neurological conditions (e.g., stroke or spinal cord/brain injury) [14]; in chronic conditions (Parkinson’s disease and Alzheimer’s disease); and, interestingly, in diabetes and atherosclerosis as well [15].

PPARγ can be activated by different natural or synthetic ligands, solely for their role in fat/glucose metabolism; among these, there are anti-diabetic drugs such as thiazolidinediones. Among the natural agonists of PPARγ, some terpenoids, flavonoids, and alkaloids [16], as well as gamma-oryzanol [17,18], are now known. The genes activated by PPARγ stimulate lipid recovery and lipogenesis in fat tissue. Several in vivo studies have demonstrated that PPARγ knockout mice did not generate adipose tissue despite being fed with a high-fat diet [19,20].

Among the mechanisms that are involved in metabolism and homeostasis, the cellular uptake of glucose and glucose transport are of fundamental importance. In particular, in humans, three families of glucose transporters are known so far: the GLUTs superfamily, the sodium-driven glucose symporters, SGLTs, and the newest, SWEETS [21]. To the GLUTs superfamily belong 14 isoforms with different tissue distributions [22]. The GLUT-4 receptor is the isoform mainly expressed by adipocytes and the most regulated by changes in the insulin plasma level: indeed, a systemic increase in insulin causes the GLUT-4 translocation from the intracellular compartments to the plasma membrane [22]. Recent research by Pujimulyani et al. has demonstrated that an ethanol extract of *Curcuma mangga* Valeton and van Zijp significantly increased the GLUT-4 gene expression, contributing to the hypoglycaemic action of the plant [23].

Recent literature evidence has also shown that, in in vivo models, GLUT-4 is present in the brain, particularly in the hypothalamus, a structure that, as is well known, plays an important role in glucose homeostasis and in energy balance [24,25,26,27].

Moreover, Leguisamo et al. [28] demonstrated in a rat model of metabolic syndrome that a reduction in GLUT-4 protein levels was accompanied by an increase in plasmatic inflammatory markers such as the cytokines IL-6 and TNF-α or the C Reactive Protein (CRP). Other studies showed indirectly the inverse correlation between GLUT-4 expression and inflammatory cytokine levels, demonstrating, for example, that natural extracts or chemical compounds could induce these opposing effects in different in vitro models [29,30]. 

The scientific literature is poor in references regarding the punctual chemical characterization of extracts of CP whole plant. According to CAS SciFindern (a tool for the database research of the Chemical Abstracts Service (CAS)), 100 articles dealing with CP have been published in the last 20 years, and in particular, 22 analysed aspects of the chemical characterization of its drugs. Of the latter 22, some deal with the evaluation of the biological activity of extracts (aqueous, alcoholic, or hydroalcoholic) obtained from CP, giving little indication of their phytochemical characterization [31,32,33]; others considered different parts of the plant or extraction methods to those considered in this study and, for the most part, those with different polarities [34]. The main secondary metabolites reported were: alkaloids such as shankhapushpine, convolamine, convoline, convolidine, convolvine, confoline, convosine, and scopoletin; β-sitosterol; ceryl alcohols; 20-oxodotriacontanol; tetratriacontanoic acids; flavonoid-kampferol; and steroids-phytosterols [1].

Therefore, this study first sheds light on the chemical fingerprinting of the medium polarity part of the whole plant, which is mainly enriched with alkaloids and sterols. Furthermore, this study aims to evaluate the effects of CP total chloroform extract and its fractions on 3T3-L1 cell adipocytic differentiation and the potential ability of these compounds to modulate lipidic and glucose metabolism through the analysis of the key genes PPARγ and GLUT-4. The importance of the research is due to the possibility to employ CP to ameliorate or better control lipid parameters and glycaemia, not only in pathological or sub-pathological conditions but also in healthy people, in order to prevent cardiovascular involvement or complications.

## 2. Materials and Methods

### 2.1. Plant Material

The whole plant of *Convolvulus pluricaulis* Choisy (CP, Batch No. 7028) was collected from Ram Bagh (Rajasthan, India), authenticated by Dr. MR Uniyal, Maharishi Ayurveda Product Ltd., Noida, India, and imported by MAP Italia. After harvesting, the whole plant was cleaned and cut into small pieces before being dried. All the samples were then ground to a fine powder and kept at −20 °C until the moment of the extractions.

### 2.2. Convolvulus pluricaulis Extraction

Fifty grams of CP whole plant was defatted using 500 mL of n-hexane (Sigma-Aldrich, Milano, Italy) in a solvent-to-sample ratio of 1:10. The sample was ultrasonicated for 50 min, and the hexane was removed by filtering. The operation was repeated with a further 500 mL of fresh solvent. Then, the plant material was thoroughly dried and extracted for 50 min by ultrasonication at maximum power with 500 mL of a 2% (*w*/*v*) solution of potassium hydroxide in ethanol. This extractive step was repeated two more times on the same portion of starting plant material to increase the final extractive yield. After filtration, the ethanolic extract was dried with a rotary evaporator (RV 10 digital, IKA^®^-Werke GmbH & CO. KG, Staufen im Breisgau, Germany). The dried extract was resuspended in 100 mL of ddH_2_O and placed in a separatory funnel, where 100 mL of chloroform (CHCl_3_; Sigma-Aldrich, Milano, Italy) was added, according to a procedure previously reported in the literature [35]. The extract was collected and another 100 mL of CHCl_3_ was added. This operation was repeated one more time. The three CHCl_3_ fractions were collected, brought to dryness, and stored at −20 °C until the further separation steps. The complete extraction process was repeated three times.

### 2.3. Flash Chromatographic Separation 

The chloroform extract was fractionated by flash chromatography using silica gel (Merck, Darmstadt, Germany) as the stationary phase and chloroform and ethyl acetate (Merck) at a ratio of 8:2 as the mobile phase. Four main fractions were collected (1, 2, 3, and 4) and evaporated to dryness. Each obtained fraction was resuspended in chromatographic grade chloroform (Merck) and analysed by gas chromatography coupled with a mass spectrometer (GC-MS) and a flame ion detector (GC-FID). 

### 2.4. GC-MS and GC-FID Analyses

The GC-MS technique was used to qualitatively analyse the extract of CP; the GC-FID was used to obtain quantitative data. The GC-MS analyses were performed by a Varian GC-3800 gas chromatograph (Palo Alto, CA, USA) equipped with a Varian MS-4000 mass spectrometer (Palo Alto, CA, USA) using electron impact and hooked to the NIST library. The GC-FID analyses were performed with a ThermoQuest GC-Trace gas-chromatograph (ThermoQuest Italia, Rodano, Italy). The gas chromatographic conditions were as follows: injector temperature 300 °C, carrier (helium) flow rate 1.2 mL/min, and split ratio 1:50. The initial oven temperature was 100 °C; then, it was raised to 320 °C at a rate of 5 °C/min and then kept constant for 20 min. One microliter of each fraction was dissolved in CHCl_3_ and injected. The mass spectrometry conditions were the same as those reported by Guerrini et al. [36]. The identification of compounds was performed by comparing their retention times and MS fragmentation pattern with those of the pure compounds, mass spectra libraries, and literature data. The extract percentage composition was calculated by the normalization method from the GC peak areas of three repeated injections, without using correction factors. 

### 2.5. Cell Cultures and Induction of Adipocyte Differentiation

The murine 3T3-L1 cell line was purchased from the American Type Culture Collection (ATCC; Manassas, VA, USA) and routinely cultured in Dulbecco’s Modified Eagle’s Medium (DMEM), supplemented with 10% foetal bovine serum (FBS), 2 mM L-glutamine, and 100 U/mL penicillin/streptomycin (all from Gibco BRL, Grand Island, NY, USA) in an atmosphere of 5% CO_2_ and 90% relative humidity at 37 °C. For the induction of the adipocytic differentiation, the 3T3-L1 cells were cultured for 3 days with DMEM containing 5% FBS, L-glutamine and penicillin/streptomycin in the presence of 1 mg/mL dexamethasone, 0.2 nmol/L insulin, and 0.5 mmol/L isobutyl methyl xanthine (all from Sigma-Aldrich, St. Louis, MO, USA), as previously described [37]. After the first 3 days of culture, the medium was replaced with DMEM supplemented with 5% FBS, L-glutamine, penicillin/streptomycin, and only 0.2 nmol/L insulin. The cells were left in these culture conditions for an additional 3 days and then monitored for adipocytic differentiation by morphologic examination and analyses of the peroxisome proliferator-activated receptor γ (PPARγ) gene expression levels. 

### 2.6. Extract and Fractions’ Dissolution for Cell Treatments

During the differentiation time, the cells were left untreated or treated with different concentrations of fractions 1, 2, and 4 and of the total extract. For this purpose, the dried compounds were dissolved in dimethyl-sulfoxide (DMSO) at concentrations that allowed them to avoid any vehicle toxicity. F1, F2, and F4 were used at different ranges of concentration, due to the distinct solubility of the compounds.

Fraction 3 was not included in the study because of its very low solubility, which makes it impossible to use for cell treatments.

### 2.7. Morphological Analysis and Oil-Red O Staining

After the whole period of differentiation and treatment, the 3T3-L1 cell cultures were observed with an EVOS inverted microscope (AMG, Advanced Microscopy Group, Mill Creek, Washington, DC, USA), and phase contrast images were taken.

The differentiated cells were also stained with Oil-Red O to highlight the cytoplasmic lipidic content. Briefly, the cells were fixed with 10% formalin for one hour, washed with 60% isopropanol, and then stained with Oil-Red O 2.1 g/L. Images were acquired with an Aperio ScanScope slide scanner by using the Aperio ImageScope v11.1.2.760 software (Leica Biosystems, Nussloch, Germany). 

### 2.8. RT-PCR Analysis

RT-PCR analysis of PPARγ and GLUT-4 mRNA modulation was performed after the whole period of differentiation in 3T3-L1 cultures left untreated or treated with fractions 1, 2, and 4 and with the total extract.

High-quality total RNA from the cells under different experimental conditions was extracted with miRNeasy Mini kit (QIAGEN, Hilden, Germany), as previously described [38]. Briefly, after genomic DNA removal with the RNase-Free DNase Set (QIAGEN), the RNA was retro-transcribed and amplified with the Express One-Step Superscript qRT-PCR Kit, universal (Thermo Fisher Scientific, Rockford, IL, USA), using the TaqMan assay technology and a QuantStudio™ 3 Real-Time PCR System (Thermo Fisher Scientific). Mouse Ppia (Mm02342430_g1) was used as the housekeeping gene. The TaqMan assays used were Mm00440940_m1 (for PPARγ) and Mm00436615_m1 (for GLUT-4). Fold changes were calculated using the 2^−ΔΔCT^ method. Cycle thresholds >35 were excluded. Control PCR samples were run without RNA.

### 2.9. Cytokines Analysis

The cytokine analysis was performed in duplicate on the culture supernatants at the end of the adipocytic differentiation, using the 23-Bio-Plex assay (BioRad Laboratories, Milan, Italy) and read on a MAGPIX instrument (Merck Millipore) equipped with the MILLIPLEX-Analyst Software (Merck Millipore), using a five-parameter nonlinear regression formula to compute the sample concentrations from the standard curves. The quality controls provided in the multiplex kits were used to validate the assay performance. For the cytokine analysis, the supernatants were always frozen and thawed only once.

The cytokines analysed were: interleukin (IL)-1α, IL-1β, IL-2, IL-3, IL-4, IL-5, IL-6, IL-9, IL-10, IL-12 (p40), IL-12 (p70), IL-13, IL-17A, eotaxin, granulocyte colony-stimulating factor (G-CSF), granulocyte-macrophage colony-stimulating factor (GM-CSF), interferon-γ, keratinocyte-derived chemokine (KC), monocyte chemotactic protein-1 (MCP-1), MCAF (monocyte chemotactic and activating factor), macrophage inflammatory protein (MIP)-1α, MIP-1β, regulated and normal T cells expressed and presumably secreted (Rantes), and tumour necrosis factor-α (TNF-α). 

### 2.10. Statistical Analysis

The results of the chemical characterization were expressed as the mean ± standard deviation (SD), while the data obtained from the biological evaluations were expressed as the mean ± standard error of mean (SEM). Statistical significance was calculated using a one-way analysis of variance (ANOVA) and Bonferroni post-hoc test correction in the case of multiple comparisons. Analysis was performed using GraphPad Prism software (version 8.4.2).

## 3. Results

### 3.1. Convolvulus pluricaulis Fractions Chemical Characterization 

**Figure 1:**
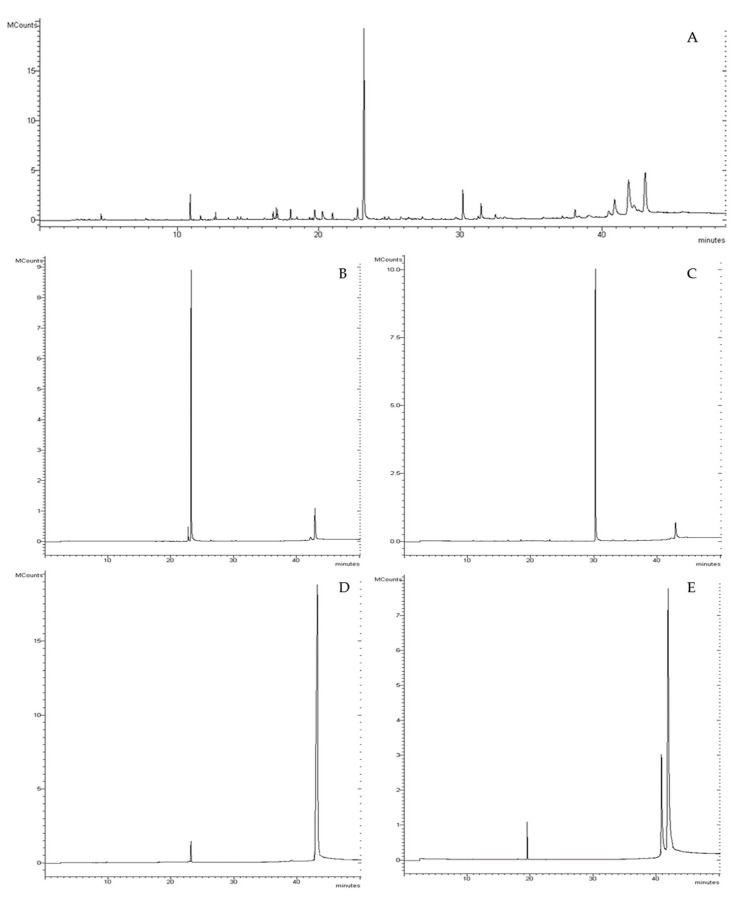
Chromatographic analysis of CP: (**A**) chromatogram of the total extract of CP; (**B**–**E**) chromatograms of fractions 1 to 4, respectively, obtained by flash chromatography.

The *Convolvulus pluricaulis* (CP) whole plant extract obtained after the alkaline ethanolic phase showed a yield of 18.23 ± 0.92%, while the complete process (after the liquid–liquid extraction) exhibited a total yield of 0.27 ± 0.01%. This latter phytocomplex was subjected to further separation by flash chromatography, obtaining four fractions, which represented 79.1% of the total extract (Figure 1). 

The fractions, named F1, F2, F3, and F4, showed a yield of, respectively, 25.20 ± 1.07, 21.57 ± 0.74, 19.14 ± 1.22, and 13.39 ± 1.02%. The chemical characterization of the total extract (CP_TOT_) and the four fractions is shown in Table 1. This study shows, for the first time, that the CP_TOT,_ derived from the whole plant, is mainly characterized by the presence of trans-phytol (vit. E precursor), by phytosterols (31.09%), of which β-sitosterol is the most abundant, and triterpenoids (26.81%), mainly represented by lupeol (17.28%). Alkaloids were not revealed. The literature did not reveal comprehensive articles concerning the chemical characterization of the whole-plant drug of CP. Sethiya et al. [39], however, report a characterization indicating the presence of β-sitosterol and other phytosterols in the whole plant. Interestingly, the presence of phytol in CP does not emerge in any scientific publication and only appears in the work of Rachitha et al. [40], where vitamin E, of which phytol is a precursor, is mentioned. After flash separation, these main compounds were divided into four fractions: fraction 1 (F1) was rich in trans-phytol, which was never identified in this drug before; fraction 2 (F2) contained the chromanone and a small percentage of the triterpenoid taraxerol, first identified in this species; fraction 3 (F3) was characterized by the content of the triterpenoid lupeol; and fraction 4 (F4) by the phytosterols abundance. Hence, this study shows, for the first time, that CP whole plant is characterized by complex phytosterol and terpenoid fractions, of which β-sitosterol and lupeol are the most abundant.

### 3.2. Effects of Convolvulus pluricaulis Extract on 3T3-L1 Adipocyte Differentiation

**Figure 2:**
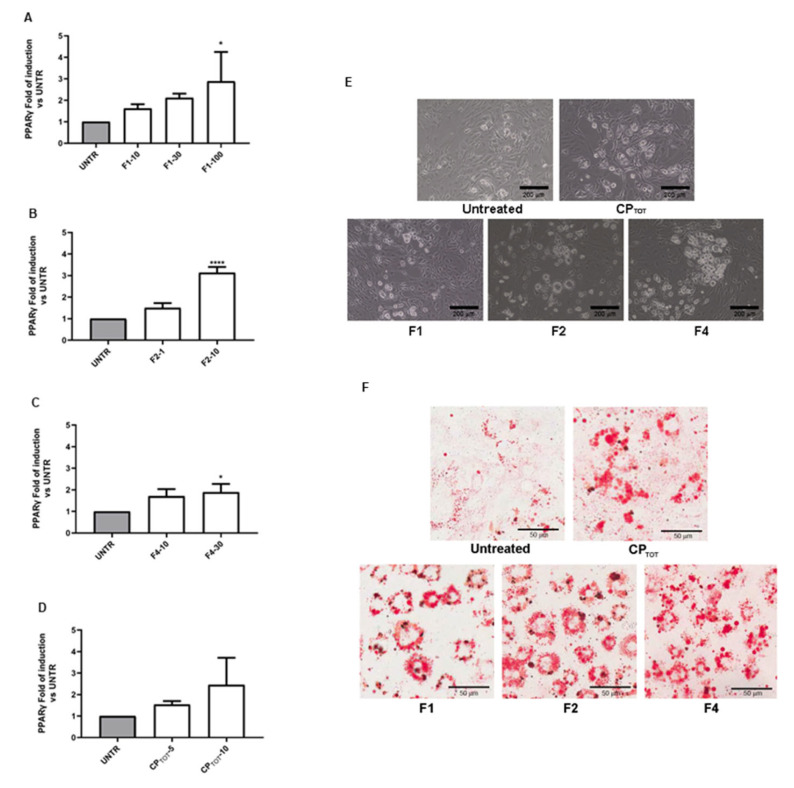
Effect of CP fractions and total extract on adipocyte differentiation. PPARγ mRNA level was evaluated, after the complete differentiation time, on 3T3-L1 cell line left untreated (UNTR) or treated with fractions 1, 2, and 4 (F1, F2, F4) or with the total extract (CP_TOT_) at different concentrations (µg/mL) (**A**–**D**). Data are reported as mean ± SEM of at least three independent experiments performed in duplicate. * *p* < 0.05, **** *p* < 0.0001. Representative contrast phase images (**E**) and Oil Red O-stained cell images (**F**) in the same experimental conditions were shown, respectively. Magnification 20×.

The effects of the CP extract and fractions were first evaluated by the analysis of the different modulations of the PPARγ gene expression in the 3T3-L1 cells at the end of the 6-days differentiation. As shown in Figure 2A–C, all three fractions induced a significant increase in the PPARγ gene expression level with respect to the untreated cells, which was indicative of the adipocyte differentiation enhancement. Of note, the maximal effect between the three fractions was obtained using F2 at the concentration of 10 μg/mL. Moreover, as expected, the total extract, especially when it was used at the concentration of 10 μg/mL, also induced an upmodulation, even if not significant, of PPARγ at the mRNA level (Figure 2D). These data were also supported by the morphological analysis of the differentiated cells observed with a contrast phase microscope (Figure 2E).

The differential effect of the three fractions on the 3T3-L1 adipocyte differentiation was also confirmed by Oil-Red O staining. At the morphological level, the increase in the multilocular lipid droplet density was very evident for all the three fractions (Figure 2F). An analogue result was obtained by treating the cells with the total extract, which clearly increased the cellular lipidic content.

### 3.3. Convolvulus pluricaulis Fractions Induced a Differential Modulation on the Insulin-Inducible Gene GLUT-4

**Figure 3:**
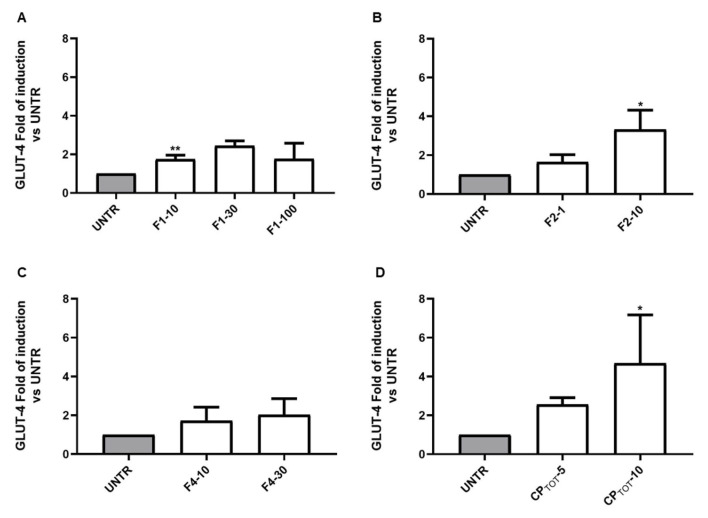
Effect of CP fractions and total extract on GLUT-4 mRNA level. GLUT-4 mRNA level was evaluated by RT-PCR on the 3T3-L1 cell line at the end of the adipocyte differentiation time and after the contemporary treatment with the indicated concentration (µg/mL) of fractions 1 (F1, in (**A**)), 2 (F2, in (**B**)), and 4 (F4 in (**C**)) or of the total extract (CP_TOT_, in (**D**)). Untreated cells (UNTR) did not receive any treatment. Results are reported as mean ± SEM of at least three independent experiments performed in duplicate. * *p* < 0.05, ** *p* < 0.01.

In the same set of experiments, the modulation of the insulin-inducible gene GLUT-4 by the different fractions was investigated. Likewise, the induction of PPARγ, as well as the GLUT-4 expression level, was significantly increased by F1 and F2 with respect to the differentiated untreated cells, which were set to 1. In particular, between the two fractions, the treatment of 3T3-L1 with F2, at the concentration of 10 μg/mL, induced the highest induction of GLUT-4 mRNA (Figure 3A,B). F4, as seen for PPARγ, was also able to induce GLUT-4 expression upregulation, but at a lower rate compared to F1 and F2 (Figure 3C). Moreover, the evaluation of the effect of the total extract on GLUT-4 revealed that this mixture was the most effective in significantly upregulating its expression level when compared to the single fractions (Figure 3D).

### 3.4. Convolvulus pluricaulis Extract Downmodulated the Release of Specific Inflammatory Cytokines

**Figure 4:**
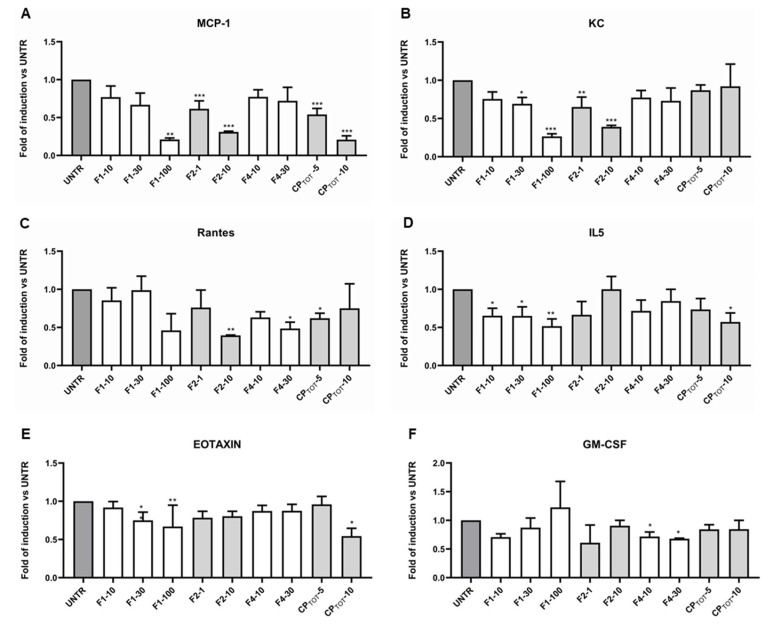
Effect of CP fractions and total extract on inflammatory cytokine release. Extracellular inflammatory cytokine release was evaluated in the culture medium at the end of adipocyte differentiation on 3T3-L1 cells left untreated (UNTR) or treated with the indicated concentration (µg/mL) of fractions 1, 2, or 4 (F1, F2, F4) or of the total extract (CP_TOT_). The following cytokines’ modulation are shown: MCP-1 (**A**), KC (**B**), Rantes (**C**), IL5 (**D**), eotaxin (**E**) and GM-CSF (**F**). Results are expressed as mean ± SEM of at least two independent experiments. * *p* < 0.05, ** *p* < 0.01, *** *p* < 0.001.

Among all of the 23 inflammatory cytokines analysed by the MAGPIX instrument, only MCP-1, KC, Rantes, IL-5, eotaxin, and GM-CSF release exhibited a relevant modulation induced by the different fractions and by the total extract. In particular, the release of MCP-1, as shown in Figure 4A, was significantly downmodulated by F1 (at the highest concentration) and F2 and by the total extract; however, it decreased in a dose-dependent manner. Moreover, the KC level was significantly downregulated by F1 and F2, but the total extract did not impair its release (Figure 4B). The extracellular level of Rantes, however, was visibly lowered by the highest concentration of all the fractions and by 5 μg/mL of total extract (Figure 4C). On the other hand, IL-5 release (Figure 4D) was significantly impaired only by F1 and by the highest concentration of the total extract, although the other fractions (excluding 10 μg/mL of F2) were able to decrease its extracellular level. Similarly, as shown in Figure 4E, the eotaxin was significantly downmodulated by the highest concentrations of F1 and of the total extract. Of note, only F4 was able to significantly decrease the GM-CSF level, even though F1 and F2 also caused a small reduction in its extracellular release (Figure 4F).

Excluding the effect on the cytokines just described, the fractions and the total extract did not show to cause any significant alteration in the release of the other cytokines analysed.

## 4. Discussion

Despite its large employment as a therapeutic agent in Ayurvedic medicine, *Convolvulus pluricaulis* Choisy (CP) whole plant extracts have not yet been fully characterized for their chemical composition. Indeed, the literature reports general references to the presence of phytosterols and flavonoids [39], mainly in the more polar extracts [40]. In this paper, newly identified molecules, such as chromanone, campesterol, lupeol, and vit. E precursors (trans-phytol and isophytol) are reported in the medium polarity extract of the CP whole plant. The pieces of evidence underline the importance of these newly identified constituents as candidate adjuvant molecules in the regulation of glucidic and lipidic homeostasis. In particular, Elmazar et al. [41] demonstrated that phytol could have an antidiabetic/insulin-sensitizing effect and that this action was strictly related to the high affinity of its derivative, phytanic acid, with PPARγ and to its ability to upregulate the transcriptional activity of PPARs/RXR heterodimers that, in turn, induce several genes involved in glycaemic and lipid homeostasis. A study performed on type 2 diabetic rat adipocytes [42] reported that the main component of F4, β-sitosterol, besides controlling hyperglycaemia and insulin resistance by upregulating GLUT-4 and PPARγ, was able to restore the normal level of proinflammatory cytokines, such as leptin, resistin, TNF-α, and IL-6, but, on the other hand, it was able to increase cytokines such as adiponectin, which acts positively on metabolism with its anti-inflammatory activity.

Moreover, triterpenoids potently ameliorate the glucidic and lipid parameters acting on PPARγ [43,44]. It was demonstrated that taraxerol, present in the F2 fraction, was involved in reversing insulin resistance induced by dexamethasone [45], and it was able to restore renal physiology in a rodent model of type 2 diabetes by lowering blood glucose and reducing the secretion of pro-inflammatory cytokines such as MCP-1, IL-1β, IL-6, and TNF-α [46].

Of note, the main component of the F2 fraction, 7-Hydroxy-3-(3,4-methylenedioxyphenyl)-4-chromanone, belongs to the flavonoid family, which is described as positively affecting glucose metabolism, potentiating, for example, the insulin-stimulated glucose uptake [47] or stimulating the translocation of GLUT-4 to the plasma membrane in 3T3-L1 adipocytes [48].

The first significant effect that we observed was the ability of chloroform CP extracts to act on PPARγ, a key regulator gene of adipocyte differentiation, upmodulating its mRNA expression. PPARγ is deeply implicated in lipid and glucidic metabolism and, for this reason, is a fundamental target of anti-diabetic drugs such as thiazolidinediones (TZDs, such as pioglitazone and rosiglitazone). These PPARγ agonists induce adipocyte differentiation and fatty acid and glucose uptake, increasing GLUT-1 and GLUT-4 expression and membrane translocation, and, as a consequence, lowering lipidaemia and glycemia [49]. Moreover, it is known that PPARγ agonists can restore insulin sensitivity and counteract inflammation. Studies in semi-knockout mice for PPARγ (PPARγ+/−) have shown that this receptor affects the release of adipokines from adipose tissue by suppressing TNF-α and IL-6 and inducing adiponectin, promoting in turn insulin sensitivity and β-oxidation of fatty acids in the liver [50]. Additionally, PPARγ inhibits hepatic lipid peroxidation, resulting in a decrease in inflammatory cytokines.

Given the importance of PPARγ agonists in diabetes therapy, it is fundamental to take into account, however, the deep side effects caused by these drugs, such as oedema, headache, heart failure, hypoglycaemia, myalgias, and increased risk of fractures [51].

In this scenario, and translating the above considerations to our in vitro model, the significant upregulation of GLUT-4 mRNA expression and the downmodulation of cytokines such as Rantes, MCP-1, and KC induced by CP, in agreement with previous studies that inversely correlate GLUT-4 expression with inflammatory markers [28,29,30], acquire particular importance. It is known, indeed, that the level of Rantes is increased in obese [52] and in diabetic people [53] and is associated with impaired glucose tolerance [52] and metabolic syndrome [54]. Analogously, MCP-1 is associated with insulin resistance, hepatic steatosis, and macrophage infiltration in adipose tissue [55]; indeed, the addition of MCP-1 to differentiated 3T3-L1-derived adipocytes decreases insulin-induced glucose uptake and the expression of specific genes such as PPARγ and GLUT-4, contributing to a pathological condition related to hyperinsulinemia and obesity [56]. KC, instead, is a key component of the inflammation of adipose tissue in obesity and in the manifestation of insulin resistance [57]. Of note, high levels of eotaxin in serum from diabetic patients have recently been reported by Okdahl et al. [58], as well as in the vitreous fluid of subjects affected by diabetic retinopathy [59], which represents an important and invalidating complication of diabetes. A similar involvement has been demonstrated for GM-CSF, whose concentrations resulted significantly elevated in proliferative diabetic retinopathy [60,61]. This clear correlation between diabetes and its complications and inflammation, calls for a continuous effort in researching alternative therapeutic approaches which are able to act in a double direction, performing an anti-inflammatory effect and lowering circulating glucose levels.

In this respect, our work demonstrated that CP, beyond the anti-inflammatory action, was also able to upmodulate the GLUT-4 mRNA expression level. It is amply accepted that increased GLUT-4 levels are directly correlated to an improved glucose uptake [62,63] and to an ameliorative/regulatory effect on glucidic metabolism via the Akt signalling pathway [64], which in turn stimulates GLUT-4 translocation on the plasmatic membrane [65,66].

Taken together, our data, although very preliminary, led us to hypothesize that there is a positive role of CP extracts in regulating glucidic and lipidic metabolism, mimicking the PPARγ agonist anti-diabetic drugs. Other studies will be essential to verify the possibility to use the CP extract as a substitute or adjuvant of PPARγ agonists, allowing a reduction in the deep side effects characteristic of this class of drugs.

## 5. Conclusions

The results presented in our study contributed first of all to a better characterization of the chemical composition of the CP extracts and, although very preliminary, allowed us to demonstrate the agonistic role of CP on PPARγ in inducing adipocyte differentiation and, more interestingly, upregulating GLUT-4 mRNA levels in our experimental model. These effects lead us to hypothesize that there is a potential beneficial effect on glycemia exerted by CP that could be exploited as an anti-diabetic therapeutic adjuvant or, at least, to better control glucose homeostasis even in healthy people, lowering the overall cardiovascular risk.

Of note, the anti-inflammatory action exerted by CP on specific cytokines such as Rantes, MCP-1, KC, eotaxin, and GM-CSF could represent an effective mechanism to attenuate or ameliorate insulin resistance and, more generally, the glucose intolerance characteristic of obesity, in addition to ameliorating people’s overall state of health.

## Figures and Tables

**Table 1:** Chemical characterization of the total extract (CP_TOT_) and of the four fractions obtained from *Convolvulus pluricaulis*.

RT (min)	Compounds ^a^	CP_TOT_	Fraction 1	Fraction 2	Fraction 3	Fraction 4
		Area % ^b^	Area % ^b^	Area % ^b^	Area % ^b^	Area % ^b^
4.63	Glyceraldehyde diethylacetal	0.38 ± 0.02				
7.77	Eugenol	0.26 ± 0.01				
10.92	2,4-bis(1,1-dimethylethyl)-phenol	1.69 ± 0.01				
11.65	Dihydroactinidiolide	0.38 ± 0.02				
12.72	1-Hexadecene	0.55 ± 0.05				
16.99	1-Octadecene	0.81 ± 0.06				
17.08	5,5,8a-Trimethyl-3,5,6,7,8,8a-hexahydro-2H-chromene	0.95 ± 0.05				
18.02	2-Pentadecanone, 6,10,14-trimethyl-	0.83 ± 0.06				
19.35	7,9-Di-tert-butyl-1-oxaspiro[4,5]deca-6,9-diene-2,8-dione	0.27 ± 0.01				
19.55	8-Hexadecenal, 14-methyl-, (Z)-	0.19 ± 0.02				1.57 ± 0.14
19.70	Methyl ferulate	1.56 ± 0.07				
20.06	Isophytol	0.26 ± 0.02				
20.26	Ethyl ferulate	1.57 ± 0.02				
20.98	1-Heptadecanol	0.62 ± 0.01				
22.74	1-Octadecanol	1.21 ± 0.03	3.42 ± 0.09			
23.20	*trans-*Phytol	20.09 ± 1.00	72.88 ± 3.62		1.62 ± 0.08	
24.65	1-Nonadecanol	0.34 ±0.01				
25.81	E,E,Z-1,3,12-Nonadecatriene-5,14-diol	0.69 ± 0.02				
27.33	4,8,12,16-Tetramethylheptadecan-4-olide	0.53 ± 0.01				
29.68	13-Tetradecen-1-ol acetate	0.77 ± 0.01				
30.18	7-Hydroxy-3-(3,4-methylenedioxyphenyl)-4-chromanone	3.51 ± 0.02		89.44 ± 0.58		
31.24	Demethylhomopterocarpin	0.81 ± 0.01				
31.47	3,7,3′,4′-Tetrahydroxyflavone	2.49 ± 0.02				
32.48	3-(2,4-Dimethoxyphenyl)-7-chromanol	1.34 ± 0.04				
37.23	Stigmasta-4,7,22-trien-3-ol	0.56 ± 0.01				
38.11	9,19-Cyclolanost-24-en-3-ol, acetate	2.05 ± 0.02				
38.39	Stigmastan-3,5-diene	1.22 ± 0.06				
40.48	Campesterol	4.22 ± 0.07				4.66 ± 0.08
40.90	Stigmasterol	8.92 ± 0.33				22.25 ± 0.81
41.89	β-Sitosterol	14.12 ± 0.23				65.13 ± 1.06
42.26	α-Amyrin	6.15 ± 0.35	2.79 ± 0.16			
42.58	Taraxerol	3.38 ± 0.12		3.61 ± 0.13		
42.95	22,23-dihydro-Stigmasterol	trace				6.40 ± 0.04
43.07	Lupeol	17.28 ± 0.17	19.15 ± 0.19		87.10 ± 0.08	

^a^ Compounds are listed in order of elution and their nomenclature is in accordance with the NIST (National Institute of Standards and Technology) library. ^b^ Relative peak areas calculated by GC-FID.

## Data Availability

Data are contained within the article.
